# Implementation characteristics that may promote sustainability of a rural physical activity initiative: examination of Play Streets through the lens of community implementers

**DOI:** 10.1186/s43058-024-00571-2

**Published:** 2024-05-02

**Authors:** Marilyn E. Wende, M. Renée Umstattd Meyer, Cynthia Perry, Tyler Prochnow, Christina N. Bridges Hamilton, Christiaan G. Abildso, Keshia M. Pollack Porter

**Affiliations:** 1 Department of Health Education and Behavior, College of Health and Human Performance, University of Florida, Gainesville, USA; 2 Department of Public Health, Robbins College of Health and Human Sciences, Baylor University, Waco, USA; 3 School of Nursing, Oregon Health & Science University, Portland, USA; 4 Department of Health Behavior, School of Public Health, Texas A&M University, College Station, USA; 5 Department of Kinesiology, College of Health and Human Sciences, Kansas State University, Manhattan, USA; 6 Department of Social and Behavioral Sciences, School of Public Health, West Virginia University, Morgantown, USA; 7 Department of Health Policy and Management, Bloomberg School of Public Health, Johns Hopkins University, Baltimore, USA

**Keywords:** Program, Sustainability, Implementation, Physical activity, Community connectedness, Active living

## Abstract

**Background:**

Play Streets, which are community-based environmental initiatives where public spaces/streets are temporarily closed to create safe, low-cost physical activity opportunities, have demonstrated feasibility and physical activity benefit in rural US areas. Yet, information is needed to identify implementation characteristics that may promote sustainability. This study examined rural Play Streets implementation characteristics that could impact sustainability from local partners’ perspectives.

**Methods:**

Sixteen Play Streets implementation team members in rural Maryland, North Carolina, Oklahoma, and Texas, USA, participated in interviews. Semi-structured in-person individual and group interviews were conducted in the fall of 2018 (after Play Streets implementation in 2017 and 2018), recorded, and transcribed verbatim. Transcripts were analyzed using iterative, content analyses. Coding frameworks were based on the Public Health Program Capacity for Sustainability Framework, and emergent themes were also identified.

**Results:**

Interviewees’ perceived characteristics for facilitating Play Streets implementation aligned with the Public Health Program Capacity for Sustainability Framework: funding stability, political support, partnerships, organizational capacity, program adaption, and communication. Interviewees also noted the importance of cultural alignment/support and the reciprocal impact of community connectedness/engagement.

**Conclusions:**

Future research should examine the reciprocal role of public health impacts, as both outcomes and factors which may influence sustainability.

**Supplementary Information:**

The online version contains supplementary material available at 10.1186/s43058-024-00571-2.

Contributions to the literature

- This study provides evidence that Play Streets have the capacity for sustainability in rural areas.
- Findings demonstrate how the Public Health Program Capacity for Sustainability Framework is useful for assessing the potential sustainability of implementation efforts.
- Reciprocal determinism and cultural alignment and support were important themes for implementation that should be considered for inclusion within sustainability frameworks and then tested and refined.

## Introduction

Emerging research investigating strategies to increase physical activity (PA) in under-resourced areas has found that interventions that use existing community resources are most effective [1–8]. Play Streets are one example of an intervention that leverages community resources to promote PA. Play Streets have been implemented internationally to address resource access and safety inequities that impact youth PA [9]. Play Streets are defined as the temporary closure of streets or other public spaces (e.g., schools, parks), that for a specified time create a safe, low-cost space for children, adolescents, and/or their families to engage in active play [10–15]. Play Streets are especially important for youth in rural communities without access to safe and/or well-maintained PA resources [13].

Play Streets have been implemented in various US and international locations, despite a small body of research evaluating PA and community benefits. For example, over 650 Play Streets have been hosted in Chicago, IL, since 2012 [9, 10], and over 350 have been hosted in Seattle, WA, since 2013 [9, 12]. Internationally, Play Streets have been implemented in many urban locations in England, Australia, Chile, and Belgium [14, 16–18]. In addition to popularity in urban spaces with varying income levels, Play Streets have been held in rural areas such as Oakland, MD, Warrenton, NC, Talihina, OK, and Cameron, TX [15, 19]. Despite recent successful Play Streets implementation, rural communities often disproportionately experience barriers to sustaining health promotion initiatives over time [20, 21]. While research has explored barriers to sustainability in urban locations [22], research is needed with rural community partners who have successfully implemented Play Streets to understand what implementation strategies they used or could use to promote sustainability.

Existing research has established frameworks to ensure public health programs can sustain activities over time [23–25]. The Public Health Program Capacity for Sustainability Framework includes domains for public health decision-makers, program managers, program evaluators, or dissemination and implementation researchers to consider when developing and implementing interventions. These domains (listed in Table 1) were drawn from an extensive literature review of community-level tobacco use, PA, cardiovascular health, diabetes, and asthma programs [24]. Sustainability capacity is defined in the Framework as “the existence of structures and processes that allow a program to leverage resources to effectively implement and maintain evidence-based policies and activities” [24], p. 2].

**Table 1:** Schell et al.’s public health program capacity for sustainability framework components (24)

**Funding stability**	Making long-term plans based on a stable funding environment
Political support	Internal and external political environment which influences program funding, initiatives, and acceptance
Partnerships	Connections between program and community
Organizational capacity	Resources needed to effectively manage the program and its activities
Program adaptation	Ability to adapt and improve to ensure effectiveness
Program evaluation	Monitoring of process and outcome data associated with program activities
Communications	Strategic dissemination of program outcomes and activities with stakeholders, decision-makers, and the public
Public health impacts	The program’s effect on the health attitudes, perceptions, and behaviors
Strategic planning	Processes that define program direction, goals, and strategies

The purpose of this qualitative study was to examine the Play Streets implementation characteristics that relate to the potential sustainability of the programming from local partners’ perspectives in four rural US communities.

## Methods

### Study setting

MRUM and KMPP conducted qualitative, semi-structured interviews with 16 local Play Streets implementers (hereafter “interviewees”) in low-income, rural (Rural–Urban Commuting Area code ≥ 4.0), and racially and ethnically diverse US communities throughout Maryland, North Carolina, Oklahoma, and Texas in the summers of 2017 and 2018 [15, 19, 26]. Interviewees represented local community organizations, including a county health department, church, cooperative extension, and Tribal health authority, and helped secure public spaces, plan the time/date strategically, recruit volunteers, and consider liability/risk management [15, 19]. No eligible interviewees refused to participate or dropped out of the study. Table 2 includes details on implementation sites in years 1 and 2, and lessons learned incorporated for year 2. Additional information on study communities and organizations is described elsewhere [26].

**Table 2:** Characteristics of Play Streets implementation sites

Location	Organization	Year 1				Year 2			Total # interviews (total number of interview participants)
		# Play Streets	# Distinct Play Streets Sites	# Lead implementers	Lessons Learned	# Play Streets	# Distinct Play Streets Sites	# Lead implementers	
North Carolina	Church	4	2	1	Need additional volunteers and implementers	0	0	1	1 (1)
Oklahoma	Tribal Health Authority	4	4	2	Plan events at locations where people tend to congregate to increase attendance	3	3	1	1 (1)
Maryland	County Health Dept	4	1	2	Increase reach to a larger community. Include more sites that are geographically representative	4	4	2	6 (13)
Texas	Extension Office	4	1	1	Need additional volunteers. Continue to advertise that the event is free in both English and Spanish	3	1	1	1 (1)

These community organizations were deemed “ready” to implement Play Streets in the summer of 2017 because they had prior experience implementing community-level programming for school-aged children and families, although they had never implemented Play Streets [15, 19]. The research team made multiple face-to-face visits to and held regular phone meetings with each community’s implementers to develop trust and foster community capacity/efficacy to organize Play Streets. To support implementation efforts, each organization received a $6000 stipend, of which at least $1000 had to be used for reusable materials (e.g., hula-hoops). Organizations relied heavily on publicly available descriptive resources from Chicago PlayStreets (www.gadshillcenter.org/playstreets.html). All procedures were approved by the institutional review boards of the referent universities. In year 2 (2018), three of the four organizations agreed to implement at least three additional Play Streets incorporating lessons learned from year 1 implementation. Each organization received $500 per Play Streets occurrence in year 2 to support needs identified after year 1 implementation.

### Data collection

Interview guides (Additional file 1) were internally developed and pilot-tested to examine implementers (interviewees) perceptions of Play Streets implementation and outcomes after a second year, including reflections on 2 years of implementation (or de-implementation), reach, intervention characteristics, changes/adaptations, lessons learned, data collection, funding, impacts, sustainability, organizational readiness and self-efficacy, and recommendations. The Public Health Program Capacity for Sustainability Framework was not used to develop the interview guide, despite there being an overlap between the interview guide and the framework.

Eligible interviewees included Play Streets implementation team members involved in years 1 and/or 2 of implementation. Semi-structured individual (*n* = 4) and group (*n* = 3 with 2 participants each; *n* = 2 with 3 participants each) interviews lasted 60–90 min and were conducted face-to-face (at Health Department, Extension, or Tribal Authority conference rooms), except for one individual interview conducted via Zoom. Multiple interviewees were invited to participate in interviews in all locations, but only some locations/teams engaged more than 1 implementer in interviews. Interviews included in this analysis occurred in the fall of 2018, after two summers of Play Streets implementation (2017–2018). No repeat interviews were carried out. Four interviews were conducted with lead Play Streets implementers (*n* = 5 interviewees), representing four organizations, with one organization having two lead implementers (Table 2). An additional five interviews were conducted with other key Play Streets implementation team members of a Health Department in rural Maryland who helped plan and put on Play Streets, identified by the lead implementer(s). Based on lessons learned in year 1 about increasing the accessibility and reach of Play Streets, Maryland implementers increased the number of Play Streets location sites from one location in year 1 to four locations in year 2 with distinct local implementation team members; thus, our sample included a large percentage of interviewees from Maryland (81.3%) (Table 2). Interviews were recorded and transcribed verbatim, and interviewees were provided a $10 gift card in appreciation of their participation.

### Analyses

A coding protocol was developed collaboratively by four researchers (MW, MRUM, CP, TP), which was informed by field notes taken during interviews. Interview transcripts were analyzed using iterative content analyses [27–29]. Coding frameworks included a priori themes based on the Public Health Program Capacity for Sustainability Framework and emergent themes (Table 3) [24, 30]. We used this Framework to inform and guide our analysis because characteristics/factors pertinent to sustainability were covered in the interviews. Specifically, two research team members (MW, MRUM) participated in an initial phase of reading through and making memos for all transcripts. As part of this phase, reciprocal determinism, defined as “a continuous reciprocal interaction between behavioral, cognitive, and environmental influences” [31, 32], and cultural support and alignment, defined as “understanding how culture and addressing cultural differences in programs and policies promote health and well-being” [33], were identified as important emergent themes outside of the Public Health Program Capacity for Sustainability Framework and added to our coding framework (Table 3).

**Table 3:** Coding framework

Code	Description
*A priori themes*	
**Funding stability**	Making long-term plans based on a stable funding environment
**Political support**	Internal and external political environment which influences program funding, initiatives, and acceptance Dimensions: - Internal: political dynamics within the Play Streets planning group - External: community-level political dynamics impacting Play Streets planning
**Organizational capacity**	The resources needed to effectively manage the program and its activities. These resources must be directly related to program sustainability/success (selective coding) and refer to within-group dynamics (compared to partnerships that are outside the group, such as in the community)
**Partnerships**	The connection between program and community This refers to connections outside the immediate Play Streets planning group. Specifically, about partnerships with community groups/members
**Program adaptation**	The ability to adapt and improve Play Streets in order to ensure effectiveness. Specifically, this refers to adaptations from year 1 to year 2 or for the current season (i.e., changes to Play Streets that already happened)
**Program evaluation**	Monitoring and evaluation of process and outcome data associated with program activities To note, this can only be formal evaluations (i.e., involve data collection*—*like step counts or scans) that promote sustainability
**Communications**	The strategic dissemination of program outcomes and activities with stakeholders, decision-makers, and the public. This does not include advertising for the event
**Public health impacts**	The program’s effect on the health attitudes, perceptions, and behaviors in the area it serves Dimensions: - *Physical—*impact on behaviors, such as ability to eat a nutritious meal or maintain physical activity. This could also include more concrete physical health outcomes if mentioned (i.e., falls, diabetes, obesity, etc.) - *Social/emotional—*impacts on community connectedness, mental health, individual social support, and any other non-physical outcomes. Can be community- or individual-level social/emotional outcomes
**Strategic planning**	The process that defines program direction, goals, and strategies. This mostly refers to planning “going forward” but can rarely include instances where planning efforts in the past are discussed. Strategic Planning now only relates to long-term efforts
*Emergent themes*	
**Cultural support/alignment**	Defined as “understanding how culture and addressing cultural differences in programs and policies promote health and well-being.” [33] Similar to the political support code, but more related to cultural climate and acceptance of Play Streets; internal and external cultural environment which influences program funding, participation, success/sustainability, and acceptance
**Reciprocal determinism**	Defined as “a continuous reciprocal interaction between behavioral, cognitive, and environmental influences.” [31, 32] Building off of community capacity work, we will code instances where community connection or other factors are crucial for the success of Play Streets (in planning stages), but also an important *outcome* of Play Streets. For example, community experiences increased connection as a result of Play Streets happening, which is also a direct result of community *coming together* to plan it - May be an overarching concept for a transcript – with different pieces coded separately (rarely coded all in one instance) ○ Community coming together to plan play streets may be coded as organizational capacity ○ Social and community outcomes of Play Streets – coded as public health impacts (with social sub-code) ○ Community-level partnerships in the planning stage and connection between different groups in community coded as partnerships

Next, four researchers (MW, MRUM, CP, TP) coded one interview using the coding framework in Table 3 for reliability purposes, with the intercoder agreement set at 80% agreement on 95% of codes. Inter-coder agreement was reached, with an average of 94.9% agreement and 80% agreement reached on 94.7% of the codes within the transcript. After ensuring high agreement, one researcher (MW) was the lead coder and coded the remaining transcripts independently. A second researcher (MRUM, CP, TP) reviewed MW’s codes to check for agreement, discrepancies were resolved through discussion, and changes were incorporated into the final coding. Table 3 provides a full list of codes and descriptions. Content analyses were completed using NVivo software.

## Results

**Fig. 1:**
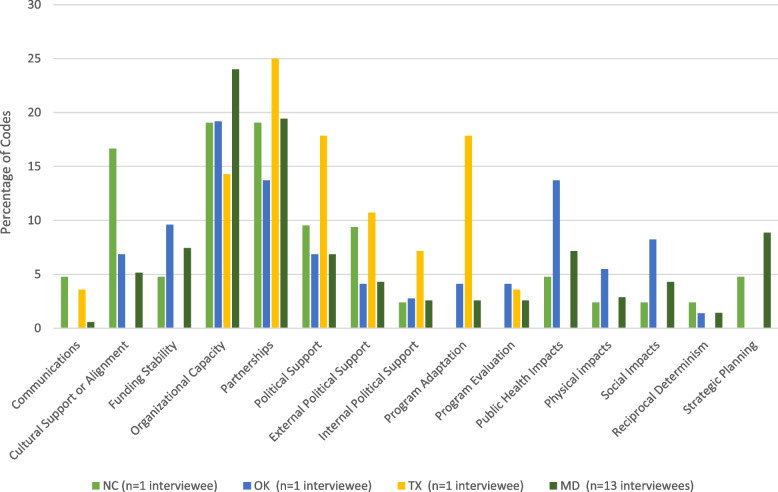
Coding matrix for interviewees in North Carolina (NC), Oklahoma (OK), Texas (TX), and Maryland (MD) (*n* = 15 interviewees)

Content analysis revealed organizational and community-level themes that have the potential to influence Play Streets sustainability and multifaceted physical and social outcomes for Play Streets attendees. The Play Streets experience included an intersection of influence between the implementers, volunteers, community organization partners, attendees, the physical environment, and the Play Streets activities. Results are presented for each of the nine themes of the Public Health Program Capacity for Sustainability Framework and the two emergent themes identified in this study: reciprocal determinism and cultural support and alignment. Figure 1 presents the count of codes for each theme according to the study site.

### Funding stability

Interviewees discussed the need to apply for grant funding to support their efforts to implement Play Streets. While grant funds are often secured by local leaders, interviewees expressed that community members themselves should be highly involved in distributing and making decisions about funding.

When we get grants, like I may get a $15,000 grant and it’s, you don’t really know what the community needs, but they do. They already have these planning groups, so you can say, ‘Just apply.’ (Maryland).

Funding for Play Streets also came from donations, health department funding, and yearly budgets associated with the interviewees’ organizations. Participants did note some barriers to acquiring donations, such as governmental changes that affect regular funding, and feeling like they could not ask other local organizations for monetary donations too many times.

I think financially grants are changing. And I think it’s because the government has changed. And so people are not real sure what decisions they’re going to say okay we’re still going to have this but we’re going to get rid of this. So people are still uncomfortable with what's going to continue to be funded. And it was really big with tribes because you’re like okay yeah we’ve got funding for the last 20 years for diabetes. But with the new administration in D.C. are we going to get our funding? (Oklahoma).

### Political support

Political support was mentioned by interviewees as both internal and external contexts which influenced Play Streets implementation. Political dynamics were noted as crucial for not only implementing Play Streets, but also sustaining them. External political support arose in terms of making sure local partners/organizations perceived Play Streets as favorable, ensuring local leaders would allow for Play Streets to be held in local parks or on main streets, and/or advertise Play Streets through pre-existing communication channels.

I think it is good and important to make sure that you do find out who is active in the community, what they are doing, and to work with those people first. Because if you don’t have the support– if we didn’t have the support of the fire department and the town and small towns can be really competitive bickering with the splintered groups, too. (Maryland).

For internal political support, interviewees mentioned the climate within the organization leading implementation that influenced Play Streets implementation and sustainability. Those planning Play Streets needed to have the motivation and drive to ensure Play Streets would be a success, which often involved ensuring there was buy-in at early planning stages. Furthermore, interviewees often had to make sure that they and associated budgetary expenses aligned with their organizational priorities (e.g., tobacco/drug prevention).

We have a health ministry… and our focus was on physical activity and nutrition as a means of managing or preventing chronic disease, so I would say again what is their focus, what are their priorities when it comes to physical activity and good nutrition and safety? …I think it needs to be something that’s important to them, because it’s going to require some sacrifice in them… organizations should have some type of already policy or commitment that’s documented, that regardless of who the leadership… some type of institutional or organizational commitment that lines up with what the Play Streets objectives are. (North Carolina).

I could maybe try to make sure there’s more physical activity… But one of our biggest priorities with these groups are that we need to get our prevention message in, which is usually an alcohol, tobacco, and other drugs. (Maryland).

### Organizational capacity

As noted in our coding framework, organizational capacity described the resources needed to effectively manage Play Streets and associated activities. Interviewees described specific resources that were needed to implement Play Streets, which included volunteers (e.g., teens, organizing/planning committee), funds (e.g., donations, grant funds), activities (e.g., inflatables), refreshments (e.g., free or for sale food/drink), amenities (e.g., bathrooms), giveaways (e.g., school supplies), and time (for multiple planning meetings).

There was a lot of planning that went into it. We met at the fire hall. And the church there was probably three or four members that came to almost every meeting. And there were members that worked that day that never came to meetings. But they informed them of what was happening. And together it was a good collaboration. We worked through a lot of bugs. And they got money for us, too, to help with food. (Maryland).

This year in order to really be successful we need more, say, buy-in or stepping up from some other church members, and I didn’t see that… I knew if I was to initiate it I wouldn’t be around to put in as much energy as we did last year, and I said when we talked earlier I didn’t see anybody else to do that, but I think it's a great opportunity, but in order for, say, our church or anybody to do it you need people in place who already are willing and can take on some responsibility. (North Carolina).

### Partnerships

Partnerships with other local community organizations were seen as crucial for facilitating Play Streets. Interviewees noted that Play Streets were most successful when they were paired with existing community events that may not have previously incorporated PA. Interviewees worked with local community organization partners to strategically couple Play Streets with a local event to ensure the greatest number of community members would attend.

Make sure that it’s a place where a lot of people that you know are coming. A vacation Bible school. A back-to-school bash. The first pep rally at the football team. You know, something where the people are already drawn there, and you’re an add-on, instead of being a standalone. (Oklahoma).

Community organization partnerships were also instrumental in other ways, such as for advertising the Play Streets, recruiting volunteers, and incorporating Play Streets activities. Community organization partners that were often noted by interviewees included local schools, churches, libraries, health-related organizations (e.g., health department, WIC), fire departments, law enforcement, or businesses. Importantly, interviewees noted that having pre-existing partnerships was crucial but also somewhat common in smaller towns.

The library they partnered with us, too. And they are starting to partner with some of our events. And they came and provided crafts for the kids at no additional charge just to be there. So, it just helps having the partners. But I think it all starts with the relationship somewhere along the line. (Maryland).

We had Healthy Texas ambassadors, 4H ambassadors, and the first one they didn’t get to go to because they were actually at their training that day, but the second and third ones they were able to come, and that was great because they could go and give the health lessons… so they were able to come and use their hours that they had to have for 4H Healthy Texas ambassador and use that for Play Street, so that was kind of nice. (Texas).

### Program adaptation

Program adaptation was defined as the ability to adapt and improve to ensure effectiveness. Interviewees noted various changes and improvements made to Play Streets to make them more successful when they implemented them a second time. Adaptations were made to improve attendance, increase PA, engage certain groups (i.e., low-income, parents, volunteers), make planning easier, ensure all Play Streets activity areas were used, and/or provide certain resources (e.g., food, handwashing station).

I think it was awesome this year. I think by having the second one I was able to really rethink everything, like what worked, what didn’t work. The one thing that I think I’m most excited about my success right on it this year was– and it’s funny, but it’s just the placement of the tents, putting it right by the fence to where basically as soon as they came in they pretty much felt that they had to come in and sign-in with me… because I felt like we talked to and we reached more people that way. (Texas).

### Program evaluation

Although collecting information on program implementation and outcomes is important for sustainability, very few participants mentioned conducting evaluations. Our research team asked lead implementers to either personally or have someone else complete video scans of each Play Streets occurrence to record the Play Streets activities implemented and visited by attendees, proportion of attendees being physically active, and the age and sex of Play Streets attendees. Some interviewees mentioned that they did not see much benefit from this evaluation, while others mentioned using it to improve their subsequent Play Streets.

I noticed at times, like, especially when I was doing the scan, that there was nobody at these different stations. And then I thought, maybe we didn’t really need those and maybe people would have been more together at events if we had less of those stations. Like the dunking booth was real popular at [town C], but everybody was standing around watching. (Maryland).

### Communications

While very few interviewees mentioned communicating or disseminating program outcomes and activities, one interviewee did mention the need for communications with local community organization partners. Specifically, attendance lists or financial reports for Play Streets and other community efforts were desired by local stakeholders and community members.

That was a big thing too this year that we did, was made sure and had people sign-in, and all we did was just have their last name and how many in their family that were attending that day or who was with their group attending that day, and it was great, because I could just email like health department, WIC– or UnitedHealthcare, WIC and health department and send them all my list. (Texas).

### Public health impacts

Public health impacts noted by interviewees included physical (e.g., PA, diabetes) and social/emotional (e.g., community connection, social support, mental health) impacts. To start, physical health impacts were mostly described as increased PA among children and families.

To me, if we don’t change our kids we don’t have any hope. If we can’t get kids up off the couch, they’re going to end up with diabetes… So we’ve got to do everything we possibly can to get people engaged that you can live a healthy lifestyle. And you do that through things like this. (Oklahoma).

While the main goal of Play Streets was to increase PA among children, interviewees seemed to view Play Streets as most successful when they engaged parents and guardians in PA with children. Interviewees noted that increasing parent/guardian PA was a successful tactic for increasing child PA.

I noticed that and I don’t know if this is really a challenge, but in [town B] in particular, that kids weren’t participating but once the adults participated, like once we set up it was the musical chairs, then the kids were all like, “Oh,” and they came and they wanted to join in then. So, they seemed like they were hesitant to do anything unless the adults– And I think it's good for adults to get in there and play. (Maryland).

Social/emotional impacts of Play Streets were also mentioned, such as the increased engagement and connection among community members. Interviewees noted that the Play Streets themselves promoted increased social engagement since it was participatory, facilitated conversation and relaxation, provided resources (e.g., food, school supplies) through community organization partnerships and donations/funding, and was directed towards all age groups.

Overall, I just think families had fun together. And the big thing in our community is even though we have a lot of activities that happen here people that live here cannot afford them like skiing. A lot of local people don’t take advantage because of the expense. Or they have to travel so far to go to different to events and things like that being rural. (Maryland).

### Strategic planning

Strategic planning is the process that defines program direction, goals, and strategies, and is often related to long term planning to improve Play Streets. Interviewees had many ideas for improving Play Streets in future years, such as increasing/decreasing the number of Play Streets activities, finding ways to increase volunteer engagement, and adapting planning processes to improve organizational capacity.

Another thing we would do is to streamline. Like if we were doing it again we would not put as many activities out… if we have volunteers to man it, fine. If not, families can do stuff by themselves. And they did. (Maryland).

I would say if you were able to develop a well-defined team and what the effort is, again like putting up a tent, “You do this piece, I’ll do this piece, and we’ll get the bigger picture done”– and we are sort of challenged with that with our church, and we find that a few people end up doing a lot of stuff… my question is “How do we get people really engaged from developing an idea then developing a project to which idea is going to be developed and then be a part of facilitating?”. (North Carolina).

Play Streets were also mentioned as opportunities to improve connections with community organization partners. Since it was crucial to engage partners and attendees during a Play Street, interviewees mentioned ways to improve these efforts in future years.

In our planning, like, for this year with After School, we’re now starting a Walk to Connect group for the kids and their parents within our program. And it was because those two kind of married together and then we’re sitting here looking at things too and I’m going, “Well, this only makes sense to do it here. And so maybe one of the Play Streets how we talked, like, you know, maybe that the youth weren’t as involved as we wanted, maybe one Play Street event is where we reach out to as many of the youth organizations as we can and set a specific date that's just for the targets, like that middle school and high school”. (Maryland).

### Cultural support and alignment

Cultural support and alignment was an emergent theme identified inductively through our coding process and was defined as the internal and external cultural environment which influences program funding, participation, success/sustainability, and acceptance. While this theme is similar to political support, it differs because it refers to the community at large, as opposed to the implementing organization or local partners/groups. This code refers to norms that are not always institutionalized or written and characterizes the “fit” between Play Streets and the local community, in terms of sharing a common goal or mission. Interviewees discussed a need to tailor Play Streets to fit with existing community initiatives or practices for them to be successful. They also discussed cultural practices that should be considered when implementing Play Streets, like the fact that parents in rural areas may prefer to leave their children at Play Streets; thus, supervision needs to be well planned.

People don’t watch their kids. They really don’t. It’s like they drop them off there. I think we live in a society around here that it’s like it’s the community’s responsibility to raise this child, in a way. And I think the more rural that you are the more people feel that way. (Oklahoma).

### Reciprocal determinism

Reciprocal determinism was an emergent theme that was also identified inductively through the coding process [24]. Interviewees noticed the reciprocal nature of community connection in the success of Play Streets but did not comment on the reciprocal nature of other sustainability themes. Communities came together using existing connections to plan Play Streets and these efforts yielded increased social connections.

I felt like, you know, by doing this I saw all the hard work and just the community just coming together. Like, that was, it was beautiful to watch and see how each community embraced their own community in love, honestly. (Maryland).

It gave a sense of community. Like there—and I would watch people—they were so excited about being there, and it gave a sense of having something to do as a community, if that makes any sense. And it wasn’t just particularly about the Play Streets, it was about the event overall. Like we’ve got a safe place for three hours, to come let our kids play. We can get their backpacks and supplies because we really don’t have the resources to do that. I can feed a kid—my kid the hotdog from the bank, you know, and then somebody can throw paint at them. And I can load them up and head home. (Oklahoma).

## Discussion

This study explored perceptions of local community organizations who successfully implemented Play Streets in rural US communities across 2 years and identified factors that may promote program sustainability. Public Health Program Capacity for Sustainability Framework themes perceived as facilitating Play Streets implementation include funding stability, political support, partnerships, organizational capacity, program adaption, program evaluation, communications, strategic planning, and public health impacts. Two additional themes emerged through inductive coding as important factors for sustaining Play Streets: cultural alignment and support and reciprocal determinism. Results demonstrate that program sustainability is a key consideration for Play Streets implementers, especially during the planning stages.

While dissemination and implementation research has increased scientific knowledge on how new scientific discoveries are translated into community settings, findings contribute to a growing body of research on effectively improving population health through sustained programming efforts [24, 34, 35]. The importance of focusing on long-term sustainability, instead of short-term implementation, has been highlighted in the Consolidated Framework for Implementation Research (CFIR) which includes adoption, implementation, and sustainability (or “sustainment”) as major implementation outcomes [25]. Both the CFIR and Public Health Program Capacity for Sustainability Framework highlight themes that are internal or external to the program under study [24, 25, 36]. The Public Health Program Capacity for Sustainability Framework highlights the importance of program sustainability, and in our study, local implementers perceived major themes from this framework as important for Play Streets implementation in four rural locations [24]. Cultural alignment and support and reciprocal determinism themes were not included in the original Public Health Program Capacity for Sustainability Framework. However, both are supported by past research in this field [37].

Implementers noted that Play Streets were more successful when they were in alignment with the cultural values of the community. This often meant doing a community assessment before planning and implementing Play Streets, to understand how each event might be tailored to the community’s needs and customs (e.g., the tendency to leave children without supervision, considering community size, and inviting members of neighboring communities). This is supported by research, showing that culturally grounded programs are more sustainable and fostered by developing partnerships with local leaders and community members [38–40], ensuring implementers share cultural values and language with local community members [33, 41], including local adaptations through iterative processes [39, 40, 42], and grounding programming efforts in concepts of health equity [33]. The CFIR states that the implementing organization’s culture must be compatible with that of the local community to increase program sustainability [25, 43]. While Play Streets implementers did not describe processes for culturally tailoring programs, they did note differences in perceived program success when the program was a better “fit” with the local community and organizations.

Findings showed that Play Streets implementation was dependent on existing social connections and had a perceived positive impact on community-level social connection. Reciprocal impacts of community initiatives have been cited in past research [32, 44–47]. One study showed that there can be reciprocal or unidirectional influences between community coalition functioning and support for prevention program implementation [44]. Emerging models for community-engaged research state that reciprocity requires an ongoing process of exchange and mutual benefit [45]. Our findings reflect this sentiment and demonstrate how community and organizational-level partnerships and social connections are crucial for program implementation. While it may be true that other sustainability themes (e.g., public health impacts, funding stability) display reciprocal determinism, it was not mentioned by interviewees in this study.

**Fig. 2:**
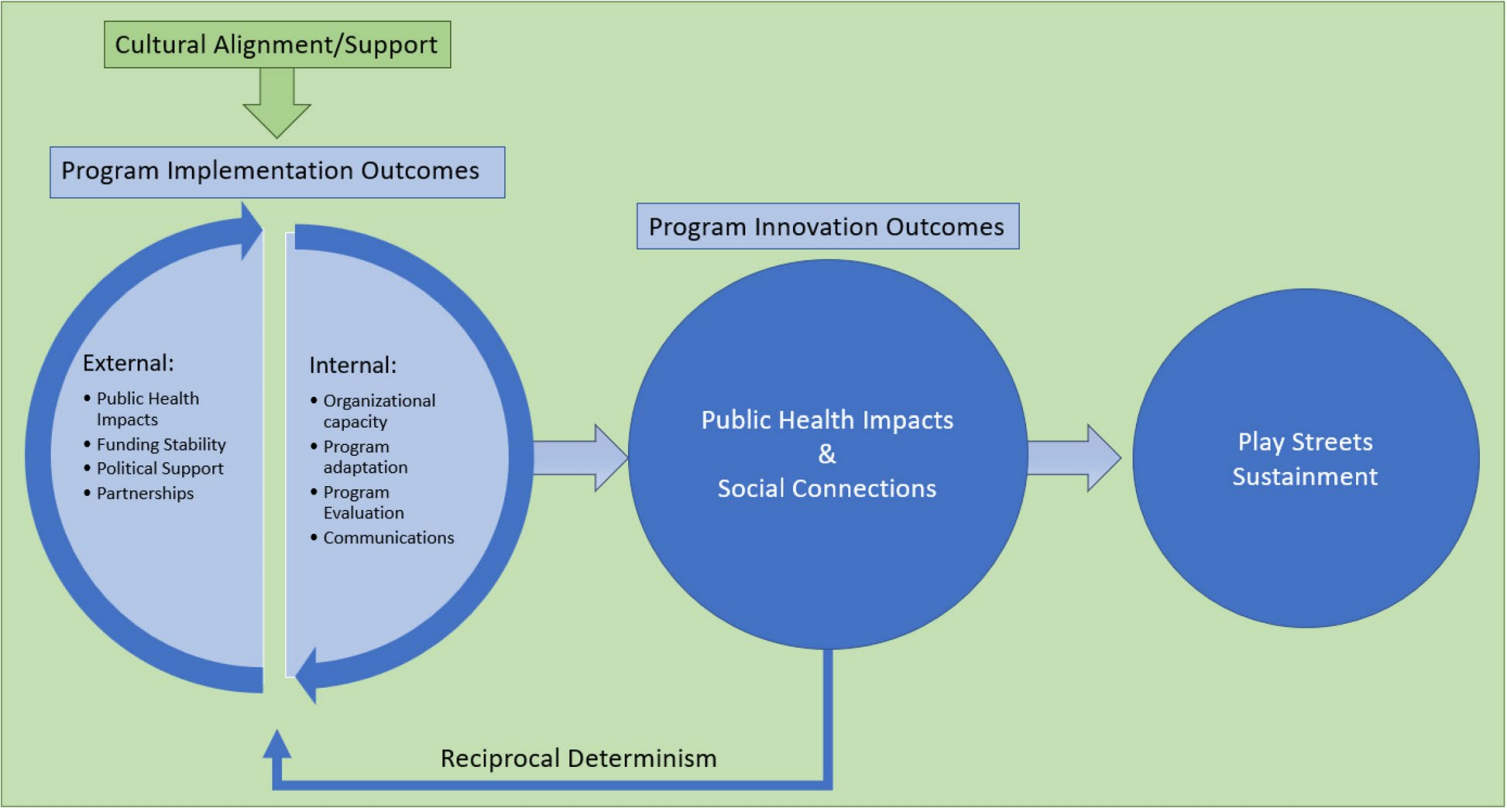
Play Streets sustainability concept map, adapted from Schell et al.’s conceptual framework for program sustainability in public health [24]

Finally, our results and Schell et al.’s existing framework helped us develop a conceptual framework to demonstrate the relationships between themes from our content analysis [24, 48, 49]. Figure 2 shows how external and internal program implementation outcomes impact program sustainability. External and internal themes may be both drivers and outcomes of Play Streets implementation and sustainability (i.e., they exhibit reciprocal determinism). Research is needed to explore the application of this conceptual framework to other Play Streets initiatives and other PA programs to verify the directions of the proposed relationships.

### Implications

Future quantitative research should examine the reciprocal role of improved social connections and whether other sustainability themes show a reciprocal relationship with program implementation. More directed interview questions may facilitate the identification of other reciprocal relationships. Research is also needed to quantitatively assess whether each of the themes identified in this study is related to actual sustained implementation of Play Streets. Finally, research is needed in additional rural settings to understand if our results and conceptual framework of Play Streets sustainability are generalizable nationally and internationally. While communities were selected to represent diverse race and ethnicities and Maryland, North Carolina, Oklahoma, and Texas are somewhat representative of the Southern region of the US, where public health and resource disparities are pronounced, they may not reflect other contexts (with different cultural norms) that may benefit from Play Streets.

Findings can be used to promote Play Streets sustainability in rural areas as they continue to gain popularity. *The Guide to Implementing Play Streets in Rural Communities* [19]*,* developed by our research team, is already being employed across the state of Louisiana and elsewhere; thus, themes related to sustainability could be incorporated into this manual to improve rural implementation efforts. Relatedly, local Play Streets implementers can measure factors related to sustainability and track progress using the reliable and valid Program Sustainability Assessment Tool [23]. Additionally, communities initiating Play Streets for the first time may use the themes identified in this paper to help design their Play Streets. Past research points to the importance of incorporating themes of sustainability during earlier planning stages to promote program success [24, 50].

### Limitations and strengths

This research has limitations. First, most of our interviewees were from Maryland because Maryland included separate implementation teams for each Play Streets occurrence and therefore had more eligible interviewees. While readers should be aware that findings showed higher representation from Maryland compared to other locations, their practice of including separate implementation teams for each Play Streets location may be a promising and sustainable model for Play Streets. Next, the individuals interviewed for this research were those who had successfully implemented Play Streets and showed readiness and capacity to do so during recruitment stages, with implementers in three of the organizations sustaining implementation across 2 years. This means that our results may not capture all important barriers or pitfalls towards Play Streets sustainability or factors that were detrimental to program success. Nonetheless, our results provide important lessons learned from rural implementers and communities that benefitted from Play Streets. Last, our interview guide was developed to capture experiences of Play Streets implementation generally and did not specifically ask about all the themes in the Public Health Program Capacity for Sustainability Framework. While our results may underestimate the importance of some themes from the Public Health Program Capacity for Sustainability Framework, interview questions did include many themes from this framework and participants were asked about factors that promote Play Streets sustainability.

This study has important strengths. First, our interview participants included local implementers who were quite knowledgeable about their community and public health programming that has been successful/unsuccessful locally. Our results provide rich detail on factors and community practices that impact Play Streets implementation and sustainability. Second, our qualitative coding and analyses were collaborative and iterative and included peer review for maximum validity and reliability. Four researchers who are considered experts in the field of PA and public health, implementation science, and behavioral programming (specifically Play Streets) contributed to our analyses, results, and commentary. Third, this qualitative research was grounded in theory to ensure results were reflective of well-established program sustainability themes. Last, results include a final conceptual framework that reflects sustainability factors perceived by implementers to be important for Play Streets implementation in rural communities. Creating this tailored and user-friendly conceptual framework is important for effective dissemination, to ensure findings can be tested, refined, and applied.

## Conclusions

This study provides evidence that Play Streets have the potential for sustainability in rural areas and demonstrates how the Public Health Program Capacity for Sustainability Framework is useful for identifying implementation efforts that may promote sustainability. We identified factors, such as reciprocal determinism and cultural alignment and support, related to Play Streets implementation that need to be considered, tested, and refined by researchers and practitioners in this field. As Play Streets continue to gain popularity in rural areas in the US and abroad, research that incorporates practice-based knowledge is crucial to ensure Play Streets can continue to yield significant public health impacts.

### Supplementary Information

**Additional file 1.** Interview guides.

## Data Availability

The datasets generated and/or analyzed during the current study are not publicly available to ensure confidentiality of participation information but are available from the corresponding author on reasonable request.
