# Supplementary material for: Implementation characteristics that may promote sustainability of a rural physical activity initiative: examination of Play Streets through the lens of community implementers
Source: Implement Sci Commun. 2024 May 2;5:48. doi: 10.1186/s43058-024-00571-2 (PMC11064337; doi:10.1186/s43058-024-00571-2)
Supplement: Supplementary file 1 — Additional file 1. Interview guides. [file 43058_2024_571_MOESM1_ESM.docx]

**Planning Committee Post Play Streets Summer 2018 Key Informant/Focus Group Guide**

*(Estimated 60-90 minutes)*

*Overall Impressions*

- What are your overall impressions of this year’s Play Streets?
- How was this years’ experience compared with last years’ experience?
- Were there any lessons that you learned from 2017 that affected how you implemented Play Streets in 2018?

*Reach*

- How did you market or advertise the Play Streets in 2018?
  - Was your approach the same or different from the one that you used in 2017?
- Did the children and families that you were expecting to attend the Play Street do so? *(Probe – get at who they expected would be there based on their outreach, marketing, etc.)*
- Think about the make-up of children and parents/guardians who attended last year (from the neighborhood, distance traveled, diversity, etc.), do you think this year’s attendees were similar to or different from last year’s attendees? How so?
- Did you notice any children or families who were at this year’s Play Streets and last year’s Play Streets? Do you have a sense of why they returned? Do you have a sense of how the experience was for them?

*Implementing Play Streets in 2018*

- What did you consider when deciding how many Play Streets you would implement in 2018?
- What influenced your decision about the duration of each Play Street?
- Describe your approach to planning this summer’s Play Streets.
  - Was your approach the same or different from the one that you used in 2017?
  - *For MD: Probe about the community meeting.*
- Think about each of the days that a Play Street occurred. Describe your approach to the day of the Play Streets*. (Probe for how you went about setup, cleanup, etc.)*
  - Was your approach the same or different from what you did in 2017?
- Did you consider access to bathrooms during the Play Streets?
  - Was your approach the same or different from what you did in 2017?
- Did you use any of the equipment that you purchased last year at all this year?
  - If yes, probe: which equipment? When? How?
- We provided you with some give-a-ways. How did you distribute those? (*Probe, gave to all kids and adults, distributed them all at Play Street, etc*.)
- How did you engage volunteers?
  - Was your approach the same or different from the one that you used in 2017?
- What were the main challenges that you encountered in putting on the Play Streets in 2018?
  - Were these challenges the same as last year or new ones?

Questions for Maryland

- Your approach to implementing Play Streets in 2018 was very different than in 2017. Can you discuss those differences and why you chose to go in a different direction?
- How did you select the five CBOs to work with?
- Why did you select the specific communities?
- Would you take this approach again? Why or why not?
- Based on your experience this year what would you recommend to other organizations interested in hosting Play Streets in multiple communities?

Questions for Texas

- You held each of the Play Streets at the same location. Would you ever consider moving the Play Streets to a different location? Why or Why not?
- How was partnering with Feeders of the Lambs for a 2^nd^ year?
- Did Feeders of the Lambs do any promoting of the days they would be partnered with Play Streets?
- Do you have any thoughts for other communities regarding partnering with summer meals programs?

Questions for OK

- This year you decided to pair one of your Play Streets with the Back-to-School event again, how did this go? What did you learn from doing this a 2^nd^ year?
- For one of the Play Streets you chose to go to a different community. Can you discuss why you chose to implement a Play Streets somewhere other than Talihina?
  - How did this work?
  - Do you feel the Play Street in a different community was successful? Why or Why not?
- Based on your experience this year what would you recommend to other organizations interested in hosting Play Streets in multiple communities?

*Data Collection*

- This year we asked you to conduct video scans of the Play Streets.
  - How was the training process for conducting the video scans?
  - How was the video scanning process?
  - Did you find any aspect of the scans challenging?
  - One way that we think the scans would be helpful is for organization to use them in “real time” so they can see how kids are using or not using the equipment and make changes for the following Play Street. While we did not do that this year, what do you think of that idea?
    - Thinking of this idea, what would be a good approach / system for this in the future?
  - After trying scans out this year, do you think you would do scans during future Play Streets if we did not ask you to do them?
  - For Tammy, Micah, James, and Kendra:
    - Did doing the scans allow you to see anything new or different about Play Streets that you did not see last year?
- This year we asked you to complete a post-Play Streets survey.
  - Did you complete the survey online or on paper?
  - Did you find any aspect of during the survey challenging?
  - Do you have anyone on your staff, or a volunteer, who would be able to look at your post-Play Streets survey responses and provide you and your planning team with feedback between Play Streets to help guide you in planning and doing your next Play Street?

*Funding*

- How did you spend the $500 that you received for each Play Street?
  - For Texas: You were able to host 3 Play Streets this summer without receiving funding from us. Will you please describe how you were able to make this work? Were you able to pay yourself back, or did you use completely different funds?
- Were you able to secure any donations?
- For MD and OK: Do you think that you could have put on the Play Streets without any funds from the grant?

*Impacts*

- Do you feel that Play Streets are making an impact? If yes, can you describe those impacts for children? Families? The community?

*Sustainability*

- Do you think Play Streets are something that you would want to continue to host in your community?
  - Why or why not?
- What would you need to continue to host Play Streets in 2019?
- What would you need to continue to host Play Streets beyond 2019?

*Self-Efficacy and Organizational Readiness*

- Did you feel prepared to put on Play Streets?
  - If yes, can you describe your previous experiences outside of Play Streets that helped you feel prepared to put on Play Streets?
  - If no, are there experiences that would have helped you feel ready to put on Play Streets?
- What do you need to do this again in the future without us?
- Are there experiences that you think would help other organizations doing this for the first time?

*Recommendations*

- Based on your two years of experience implementing Play Streets what would you recommend to other communities that are doing this for the first time?
- Do you have any new tips for others planning Play Streets in the future based on your Play Streets this year?

*Conclusions*

- Is there anything else that you want to share with us about implementing Play Streets in 2018?

Questions for North Carolina

*De-Adoption*

- You decided not to implement Play Streets in 2018. Can you please tell us what led to this decision?
- What specifically would you have needed to implement Play Streets in 2018? (Probe for people, organizations, resources, etc.)
- Did you use any of the equipment that you purchased last year at all this year?
  - If yes, probe: which equipment? When? How?
- Last year we provided you with some give-a-ways. Do you still have those?

*Re-Adoption and Sustainability*

- Do you think Play Streets are something that you would want to host in your community again in the future?
  - Why or why not?
- What would you need to continue to host Play Streets in 2019?
- What would you need to continue to host Play Streets beyond 2019?

Recommendations

- After putting on Play Streets for one year, what would you recommend to other communities that are doing this for the first time?

*Conclusions*

- Is there anything else that you want to share with us about implementing Play Streets?
